# Calculation of accurate small angle X-ray scattering curves from coarse-grained protein models

**DOI:** 10.1186/1471-2105-11-429

**Published:** 2010-08-18

**Authors:** Kasper Stovgaard, Christian Andreetta, Jesper Ferkinghoff-Borg, Thomas Hamelryck

**Affiliations:** 1 The Bioinformatics Centre, Department of Biology, University of Copenhagen, Denmark; 2 Department of Electrical Engineering, Technical University of Denmark, Lyngby, Denmark

## Abstract

**Background:**

Genome sequencing projects have expanded the gap between the amount of known protein sequences and structures. The limitations of current high resolution structure determination methods make it unlikely that this gap will disappear in the near future. Small angle X-ray scattering (SAXS) is an established low resolution method for routinely determining the structure of proteins in solution. The purpose of this study is to develop a method for the efficient calculation of accurate SAXS curves from coarse-grained protein models. Such a method can for example be used to construct a likelihood function, which is paramount for structure determination based on statistical inference.

**Results:**

We present a method for the efficient calculation of accurate SAXS curves based on the Debye formula and a set of scattering form factors for dummy atom representations of amino acids. Such a method avoids the computationally costly iteration over all atoms. We estimated the form factors using generated data from a set of high quality protein structures. No *ad hoc *scaling or correction factors are applied in the calculation of the curves. Two coarse-grained representations of protein structure were investigated; two scattering bodies per amino acid led to significantly better results than a single scattering body.

**Conclusion:**

We show that the obtained point estimates allow the calculation of accurate SAXS curves from coarse-grained protein models. The resulting curves are on par with the current state-of-the-art program CRYSOL, which requires full atomic detail. Our method was also comparable to CRYSOL in recognizing native structures among native-like decoys. As a proof-of-concept, we combined the coarse-grained Debye calculation with a previously described probabilistic model of protein structure, TorusDBN. This resulted in a significant improvement in the decoy recognition performance. In conclusion, the presented method shows great promise for use in statistical inference of protein structures from SAXS data.

## Background

The fast progress of large scale gene sequencing projects has lead to a rapid increase in the amount of known protein sequences, extending the gap between known sequences and known structures [1]. High-resolution methods have successfully been applied to resolve the structure of many proteins at the atomic level but the class of experimental conditions to which they can be applied is limited by the crystallization process for X-ray crystallography and protein size for Nuclear Magnetic Resonance spectroscopy (NMR).

These limitations can be overcome by turning to different low resolution structure determination methods. Small Angle X-ray Scattering (SAXS) [2-4] is a well established low resolution method that relies on an isotropical 1-D description of the excess electron density of the sample versus the surrounding environment. Recently, automated methods for high-throughput SAXS analysis of bio-molecules have been developed [5,6], opening the prospect of structure determination on a genomic scale from SAXS experiments. SAXS data provide information on the structure of a protein in solution, but the information content is small compared to X-ray crystallography or NMR data due to the inherent ambiguity arising from spherical averaging. This means that SAXS data only provides structural information at low resolution; additional model constraints are therefore typically needed to assist the structural interpretation.

Early SAXS structure determination methods were based on *ab initio *shape determination using spherical harmonics expansions [7]. These methods provide good computational efficiency, at the cost of limitations in accuracy for complex shapes; for instance for proteins with internal cavities [8]. Another modeling approach has been to fit the scattering curve using a gas of "dummy beads". This is done using conformational searches by a genetic algorithm in DALAI_GA [9] or simulated annealing in DAMMIN [10] and its optimized implementation DAMMIF [11]. A higher resolution approach is found in the GASBOR program [12], where a SAXS curve is fitted using a packed assembly of spheres in a pseudo-Cα chain. This program does not use amino acid sequence information, but does enforce a realistic packing density for the Cα atoms. In GASBOR, the scattering intensity is calculated using the Debye formula while simulated annealing is used for searching the conformational space. Other recent structure prediction methods, such as the ORNL [13] and IMP [14] programs, utilize the SAXS curve in the form of an extra energy term. Since these methods are non-probabilistic, the weight that scales the SAXS energy with respect to the other energy terms must be chosen heuristically [15,16].

According to the Bayesian probability calculus, the conditional probability of an event given some data depends on the *likelihood *(which brings in the data), multiplied by the *prior distribution *(which brings in the knowledge regarding the event prior to observing the data) [17]. If experimental data *D *is used to infer the structure *X *of a protein with a known primary sequence *A*, this results in the following expression:

(1) P(X|D,A)∝P(D|X,A) P (X|A)

Such an approach was used by Rieping *et al*. [15] for inferential structure determination using NMR data. The likelihood function *P*(*D*|*X*, *A*) accounts for the experimental data and quantifies the probability of observing data *D *given a protein structure *X *with sequence *A*. The prior, *P*(*X*|*A*), on the other hand accounts for general knowledge about protein structures with a given amino acid sequence [15,18-21]. In our case, the data *D *is the experimentally measured scattering curve *I *resulting from a SAXS experiment.

For the evaluation of the likelihood, it is necessary to compute the probability of the scattering profile, *I*, given a proposal structure, *X*. This work therefore focuses specifically on the calculation of the theoretical SAXS scattering curve *I *'(*X*, *A*). The likelihood can then be calculated by evaluating the discrepancy - in a probabilistic way - between the experimental curve *I *and the calculated curve *I *'(*X*, *A*). Both the curve calculation and evaluation of discrepancy must be reasonably fast in order to be useful for macromolecular structure determination. We used the well-known Debye formula [22] for calculating the scattering curve, combined with a coarse-grained representation of protein structure in order to comply with the speed requirement. In such a coarse-grained representation, certain groups of atoms are represented by one scattering body or *dummy atom *[9,12,23]. The coarse-graining thus avoids a costly iteration over all atoms (see Equation 2 below for details). The main goal of this study is therefore to obtain good point estimates of the form factors for these dummy atoms.

To illustrate and evaluate the potential of this approach in statistical inference of protein structure from SAXS data, we also perform two decoy recognition experiments (see Methods). In both cases, we use SAXS curves calculated from the native structure by the program CRYSOL as "experimental" data; the goal is to identify the native structure among a set of decoys by using the experimental data. In the first experiment, we use a simple likelihood function based on the SAXS curves calculated by our coarse-grained Debye method combined with a uniform prior. In a second experiment, we instead incorporate a probabilistic model of local protein structure as the prior distribution.

## Results and Discussion

### Coarse-grained protein models

**Figure 1:**
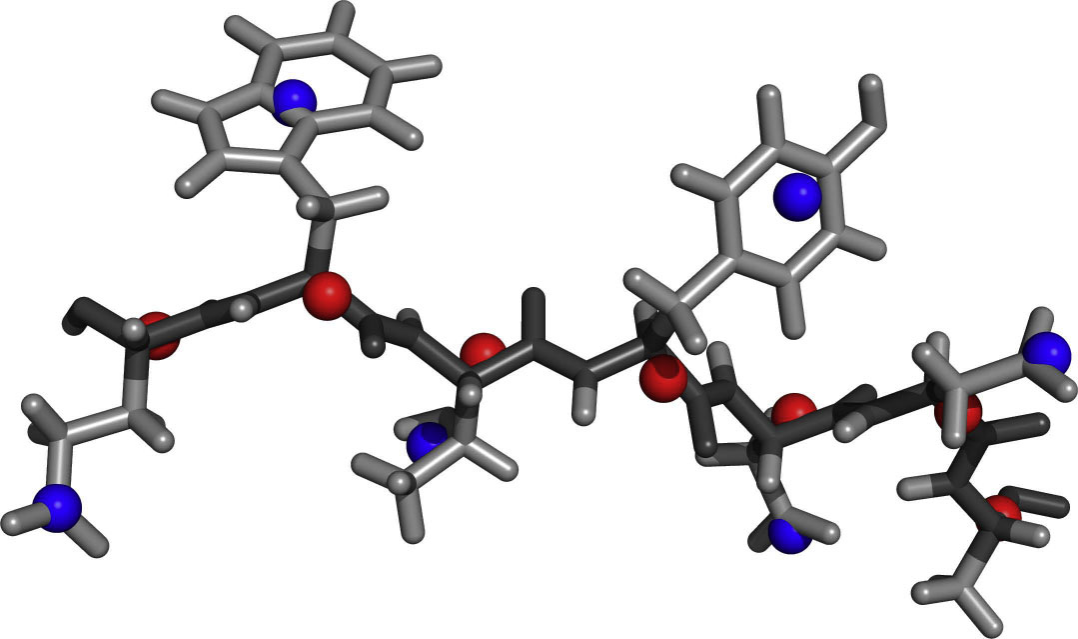
**Coarse-grained model of protein structure**. Example of a protein backbone stretch (dark gray) with side chain atoms (light gray), and a corresponding coarse-grained model. The positions of the corresponding dummy atoms for the backbone (red spheres) and side chains (blue spheres) are shown. This representation is used in the two-body model in the article. The figure was made using PyMOL [40].

We used two coarse-grained models of protein structure, in which the amino acids were represented by one and two scattering bodies (here called *dummy atoms*), respectively. In the two-body model, the amino acids - with the exception of glycine and alanine - were represented by two dummy atoms; one representing the backbone, and the other representing the side chain. The dummy atoms were placed at the respective centers of mass (see Figure 1). Exceptions were made for the representation of glycine and alanine due to their small size; in both cases, one dummy atom represents the whole amino acid. For the other 18 amino acids, a side chain specific dummy atom was combined with the generic, backbone dummy atom. As a result, a total of 21 form factors needed to be estimated for the two-body model: one for alanine, one for glycine, one for the backbone and 18 for the remaining side chains. For the one-body model, we used a straightforward approach with one dummy atom that is placed at the center of mass. Hence, 20 form factors need to be estimated; one for each amino acid type. The one-body model results in roughly half the number of scattering bodies as compared to the two-body model for a given protein.

### Calculation of the SAXS curves

The observed data in a SAXS experiment is a one-dimensional intensity curve, *I*(*q*), where *q *= 4π sin(*θ*)/λ is the scattering momentum, λ is the wavelength and 2*θ *is the scattering angle. The calculation of a theoretical SAXS scattering curve from structure is based on the well-established Debye formula [22]:

(2) $$I(q)=\sum_{i=1}^{M}\sum_{j=1}^{M}F_{i}(q)F_{j}(q)\frac{\sin(q\cdot r_{ij})}{q\cdot r_{ij}}$$

where *F*_*i *_and *F*_*j *_are the scattering form factors of the individual particles *i *and *j*, and *r*_*ij *_is the Euclidean distance between them. The summations run over all *M *scattering particles.

Since an average amino acid has around eight heavy atoms, and considering the pairwise character of the summation in Equation 2, it can be expected that a coarse-grained protein model with one or two scattering bodies per amino acid can lead to a computational speed-up of more than an order of magnitude (see Methods).

### Estimation of scattering form factors

The estimation of the form factors was carried out using artificial SAXS curves generated by the state-of-the-art program CRYSOL [24]. We used a set of 297 high resolution crystal structures from the Protein Data Bank (PDB) [25].

Atomic scattering form factors are continuous functions of the scattering momentum *q*; the same can be expected for the coarse-grained form factors. In order to render the estimation of the 20 (for the one body model) or 21 (for the two body model) form factors tractable, we discretized the problem by considering resolution bins. We divided the relevant scattering momentum interval - ranging from 0 to 0.75 Å^-1 ^- into 51 discrete bins, with a width equal to 0.015 Å^-1^. Our strategy was to obtain a point estimate for each of the 20 or 21 form factors in each of the *q*-bins, resulting in a total of 1010 and 1071 parameters for the one and two body models, respectively. We will denote the vector of form factor values for a specific *q*-bin as ${\overset{ª}{F}}_{q}$. Our scheme is to sample form factor values from a suitable posterior distribution for each bin, then calculate a point estimate from the obtained samples. We start with the classic Bayesian approach, and consider the following posterior distribution:

(3) $$P({\overset{ª}{F}}_{q}|I_{q},X)\propto P(I_{q}|{\overset{ª}{F}}_{q},X)P(X|{\overset{ª}{F}}_{q})P({\overset{ª}{F}}_{q})$$

where ${\overset{ª}{F}}_{q}$ is the 20- or 21-dimensional form factor vector for bin *q *and *I*_*q *_is the intensity calculated by CRYSOL at a given *q*-bin for a certain structure *X*. The approach will be generalized to multiple structures below. We assume conditional independence between the structure *X *and the form factor vector ${\overset{ª}{F}}_{q}$, that is, *P*(*X *|${\overset{ª}{F}}_{q}$) = *P*(*X*), and a uniform density for the prior *P*(${\overset{ª}{F}}_{q}$). To evaluate the likelihood *P*(*I*_*q*_|${\overset{ª}{F}}_{q}$, *X*) - the probability of the data *I*_*q *_given the form factor vector ${\overset{ª}{F}}_{q}$ - we use the following strategy. Applying ${\overset{ª}{F}}_{q}$, we calculate the scattering intensity ${I^{\prime }}_{q}({\overset{ª}{F}}_{q},X)$ for the given structure *X *using the Debye formula (Equation 2) and evaluate the difference between the two intensities. The likelihood is thus expressed as:

(4) $$P(I_{q}|{\overset{ª}{F}}_{q},X)=P(I_{q}|{I^{\prime }}_{q}({\overset{ª}{F}}_{q},X))$$

In the following, ${I^{\prime }}_{q}$ will be used as a short notation for ${I^{\prime }}_{q}({\overset{ª}{F}}_{q},X)$. In order to calculate the likelihood, *I*_*q *_was interpreted as a sample from a Gaussian distribution where the mean is given by ${I^{\prime }}_{q}$:

(5) $$P(I_{q}|{I^{\prime }}_{q})=N(I_{q}|{I^{\prime }}_{q},\sigma_{q})$$

with σ_*q *_being the standard deviation. The standard deviation σ_*q *_was set to a value that is typically observed in real experiments (see Methods). For multiple structures, the likelihood function simply becomes a product of Gaussian distributions:

(6) $$P(I_{q,1},\ldots ,I_{q,N}|{\overset{ª}{F}}_{q},X_{1},\ldots ,X_{N})=\prod_{i=1}^{N}N(I_{q,i}|{I^{\prime }}_{q,i},\sigma_{q})$$

where *N *is the number of structures in the training set, ${I^{\prime }}_{q,i}$ is the calculated scattering intensity curve for structure *X*_*i *_using ${\overset{ª}{F}}_{q}$ and *I*_*q,i *_is the intensity as calculated by CRYSOL from structure *X*_*i*_. Using this probabilistic model, it becomes possible to sample form factor vectors from the posterior distribution for a given bin.

The form factor vectors are sampled from the posterior distribution for each *q*-bin using a generalized Markov chain Monte Carlo (MCMC) method as implemented in the Muninn program [26].

### Form factor estimates

**Figure 2:**
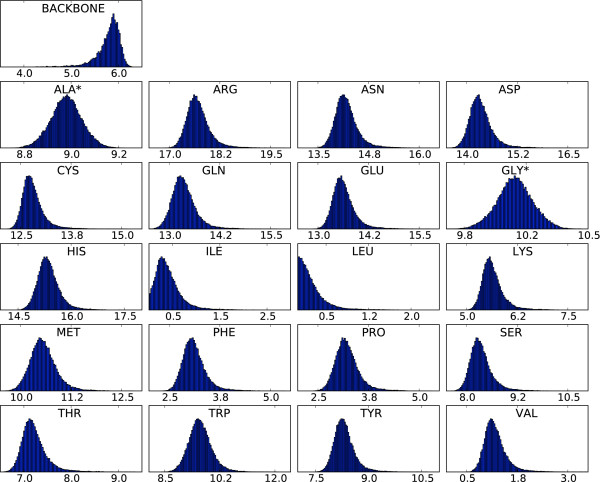
**Distributions of the components of the form factor vector for q = 0**. An asterisk indicates that this form factor describes both the backbone and side chain atoms of the residue. Note that different scales are used on the *X*-axes. The scale on the *Y*-axis shows the percentage of observations.

**Figure 3:**
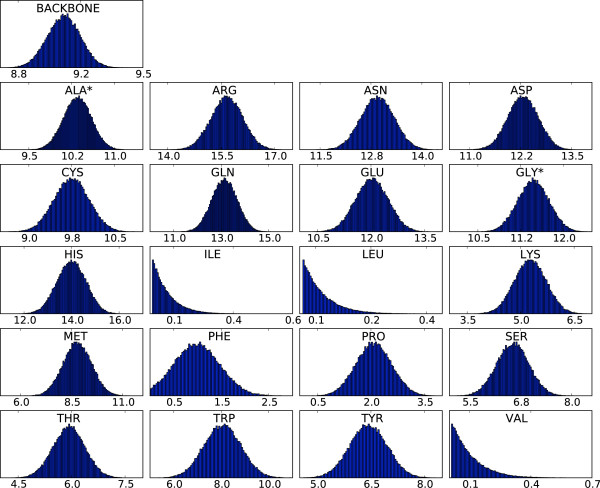
**Distributions of the components of the form factor vector for q = 0.3**. An asterisk indicates that this form factor describes both the backbone and side chain atoms of the residue. Note that different scales are used on the *X*-axes. The scale on the *Y *-axis shows the percentage of observations.

**Figure 4:**
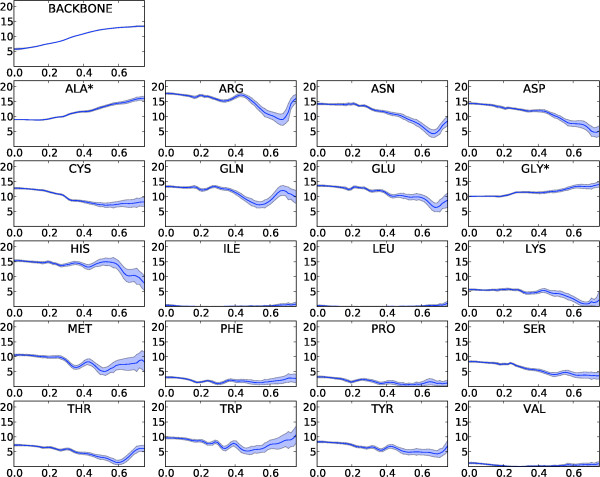
**Form factors for the two-body model**. Mean (dark blue curve) and standard deviations (blue areas) for the backbone (top left) and the 20 sidechain form factors (*Y*-axis) as a function of *q *(*X*-axis). An asterisk indicates that this form factor describes both the backbone and side chain atoms of the residue.

**Figure 5:**
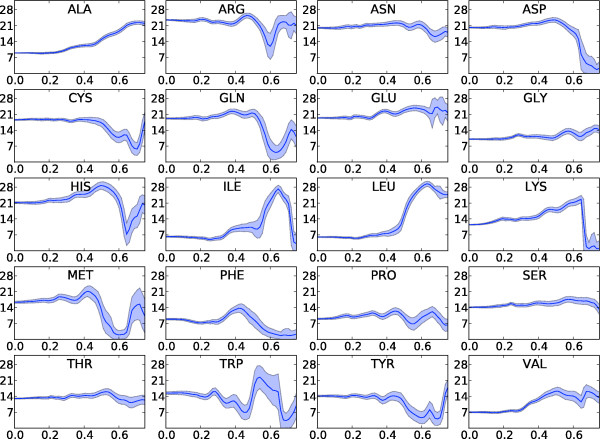
**Form factors for the one-body model**. Mean (dark blue curve) and standard deviation (blue area) of the 20 single dummy atom form factors (*Y*-axis) as a function of *q *(*X*-axis).

**Figure 6:**
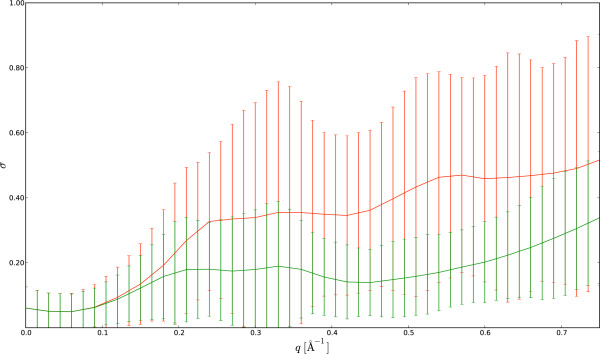
**Overall accuracy of the SAXS curve calculations**. The deviations between the SAXS curves calculated by CRYSOL and by the coarsegrained Debye methods as as function of the resolution, *q*, are shown in units of standard deviations. Mean and standard deviations are plotted in red for the one-body model and in green for the two-body model.

The resulting distributions for two *q*-bins are shown in Figures 2 and 3 (two-body model). From these distributions, it is clear that some side chains are less determining for the scattering curve than others; the hydrophobic side chains of leucine, isoleucine and valine only contribute marginally at low resolution. These amino acids are most often buried in the hydrophobic core; as a result, their contributions to the scattering intensity for low *q *values - which is mostly determined by the protein's external shape - are rather small. The final step in the estimation is to obtain the point estimates for the form factor vectors from the samples; this is done by computing the centroid of these samples (see Methods), which is an attractive point estimator for high-dimensional problems [27]. The resulting form factor curves as a function of *q *for the different amino acids and the generic backbone are shown in Figure 4. In all cases, the resulting curves are smooth in *q*. Since the estimation of the form factors has been carried out independently for each *q*-bin, the observed continuity testifies to the efficiency and consistency of the MCMC procedure. The 20 form factors for the one-body model are shown in Figure 5. Although using only one dummy atom per amino acid is computationally attractive, it comes at the cost of a significantly lower accuracy, except for very low resolutions (see Figure 6). The difference is particularly significant in the central part of the *q*-range, which is of the highest interest for structure prediction [28]. Therefore, we focus on the two body model in the rest of this article.

### Evaluation using a test set

**Figure 7:**
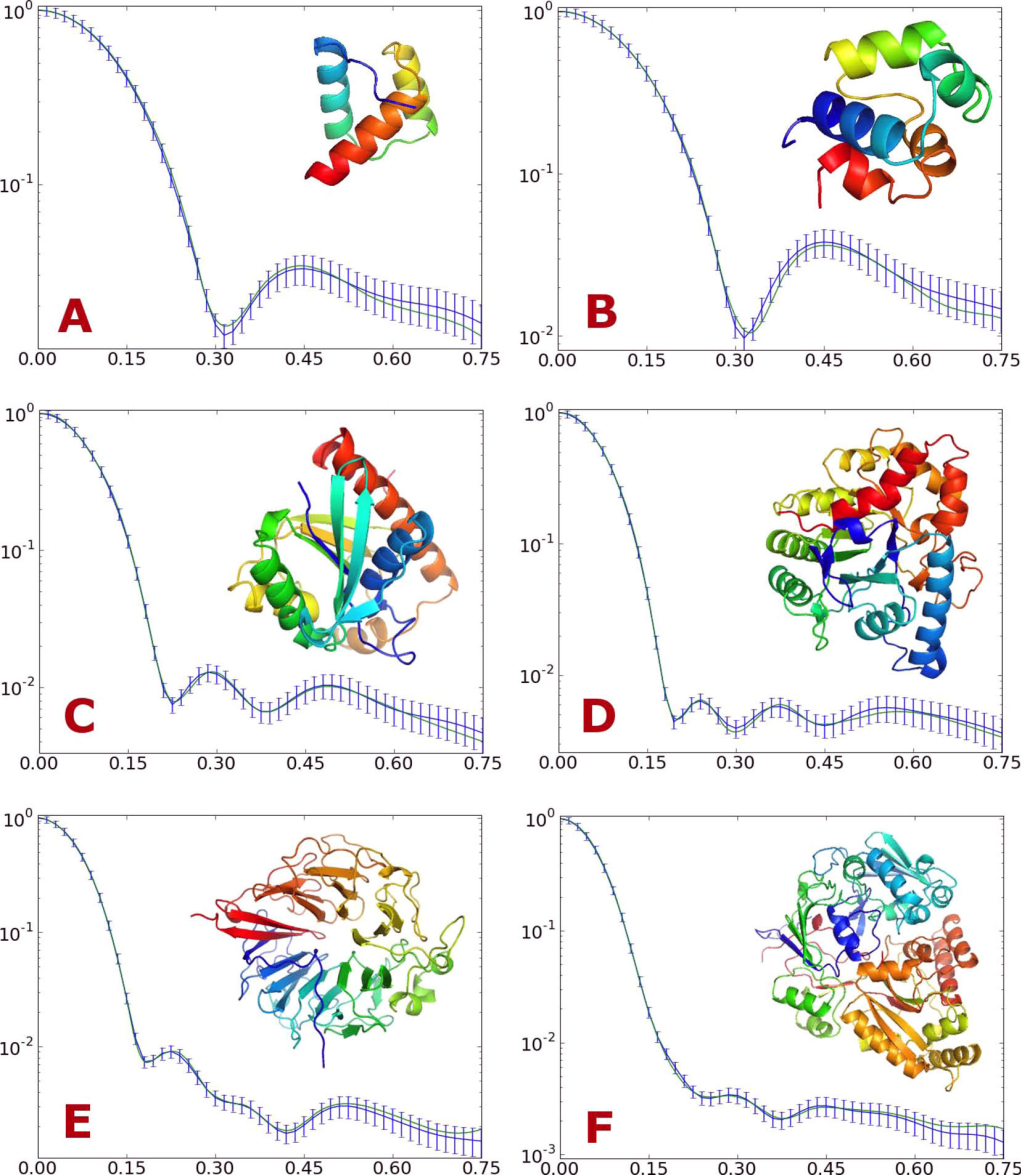
**SAXS curves for six example proteins**. Comparison of *I*(*q*) calculated by CRYSOL from the all atom structure (blue) and by the two-body model (green). Error bars indicate the simulated "experimental" error for each bin. The example proteins are: (A) 1ENH 54 residues, (B) 2CRO 71 residues, (C) 2PTH 193 residues, (D) 1PTA 327 residues, (E) 1A12 (chain A) 401 residues and (F) 1JET 520 residues.

The estimated form factors were assessed by calculating scattering curves for fifty proteins that have low sequence similarity with the proteins in the training set (sequence similarity below 25%). The calculated curves were compared to curves generated by CRYSOL, which uses full atomic detail. The results are shown in Table 1. The dissimilarities between the curves are quantified by a χ^2 ^measure, *S *in the table, which is scaled by the standard deviations that were used in the training of the form factors (see Methods for details). Since the errors are within the usual magnitude of the experimental errors [9,10,14,29], our results are in excellent agreement with the CRYSOL predictions. Scattering curves for six proteins of various sizes are shown in Figure 7.

**Table 1:** Accuracy of the SAXS curve calculation for the individual structures.

PDBcode	Chain	Length	Rg	*S*
1HCR	A	52	6.92	0.504
1TGS	I	56	6.25	0.137
1TGX	A	60	6.84	0.158
1ISU	A	62	6.02	0.203
1BF4	A	63	6.44	0.223
1PCF	A	66	8.33	0.160
1B3A	A	67	7.27	0.122
1ATZ	A	75	7.24	0.214
1DP7	P	76	7.35	0.237
3HTS	B	82	7.02	0.286
3EIP	A	84	7.35	0.233
2BOP	A	85	7.90	0.122
1LMB	4	92	7.88	0.235
1FLT	Y	94	7.55	0.132
1DIF	A	99	7.82	0.260
1IIB	A	103	7.38	0.228
1CMB	A	104	8.35	0.116
256B	A	106	8.37	0.245
1EVH	A	111	7.78	0.180
1DPT	A	117	8.56	0.194
1FLM	B	122	8.26	0.118
2BBK	L	124	8.05	0.317
1NWP	A	128	7.88	0.179
1BBH	A	131	9.18	0.161
1AQZ	A	142	8.48	0.208
1A3A	D	144	8.15	0.230
1M6P	A	146	8.70	0.141
2TNF	A	148	9.71	0.252
1ELK	A	153	8.62	0.369
1NBC	A	155	8.53	0.262
1DPS	D	156	9.82	0.272
1PHN	A	162	10.48	0.204
1C02	A	166	9.60	0.230
1YTB	A	180	11.73	0.190
1BEH	B	183	8.78	0.204
1ATL	A	200	9.30	0.267
1BSM	A	201	10.05	0.191
1YAC	B	204	9.80	0.248
6GSV	B	217	10.11	0.203
1AUO	A	218	9.34	0.137
1QL0	A	241	9.59	0.165
1CYD	A	242	10.13	0.287
1TPH	1	245	9.87	0.169
1A28	B	249	10.39	0.300
1C90	A	265	10.30	0.142
1AQU	A	281	10.73	0.177
1BF6	B	291	10.21	0.294
1FTR	A	296	12.14	0.295
4PGA	A	330	11.67	0.217
1CZF	A	335	11.43	0.241

*S *is the difference between the curves resulting from the two-body model and CRYSOL in units of "experimental" standard deviations. Rg is the radius of gyration computed from the pdb file coordinates.

### Protein decoy recognition

In order to investigate the utility of the coarse-grained model in inferential structure determination [15,16], we carried out a decoy recognition experiment (see Methods). As previously discussed, the Bayesian approach to this problem employs the posterior probability distribution. The posterior probability distribution *P*(*X*|*I*) is proportional to the product of the likelihood *P*(*I*|*X*) and the prior probability *P*(*X*). Below, we first test the model by combining the likelihood function with a uniform prior and subsequently with a suitable prior probability distribution, *P*(*X*|*A*). The performance of decoy recognition experiments is commonly evaluated using the Z-score. The Z-score is defined as the difference between the score of the native conformation and the average score of all conformations belonging to that decoy set, divided by the standard deviation [30]. Ideally, the native structures have the lowest energy. For this experiment we used a decoy set from TASSER [31].

In the first test, the likelihood was used to assign an energy to the decoys, and a corresponding Z-score was calculated. The results were compared to Z-scores obtained using CRYSOL (see Table 2). Strikingly, the coarse-grained Debye method is generally as good as CRYSOL in identifying the native structure among the decoys. In some cases our method even performs better than CRYSOL; the coarse grained approach is quite likely less sensitive to differences on the atomic scale.

**Table 2:** Decoy recognition Z-scores for CRYSOL and the two-body model.

PDB code	Chain	CRYSOL	Debye	Debye+TorusDBN
1A19	A	-1.55	-1.55	-1.83
1A7X	A	-1.83	-1.88	-2.19
1AAC	A	-1.24	-1.16	-1.75
1AAZ	A	-1.45	-1.50	-1.90
1AB1	A	-1.26	-1.36	-1.75
1AFC	A	-1.26	-1.26	-1.39
1AG6	A	-1.50	-1.77	-2.65
1B9W	A	-2.46	-2.50	-2.90
1BB9	A	-1.02	-1.43	-1.54
1BDY	A	-2.05	-2.23	-2.71
1BE9	A	-1.90	-2.16	-2.82
1BEF	A	-1.81	-2.44	-2.26
1BHO	1	-1.34	-1.83	-2.25
1BHP	A	-1.58	-1.63	-1.94
1BIK	A	-1.82	-1.81	-2.10
1BJA	A	-2.52	-2.57	-3.07
1BNL	A	-1.07	-1.59	-1.75
1BTN	A	-1.14	-2.51	-2.78
1BUN	B	-1.23	-3.44	-3.51
1BVN	T	-1.57	-2.36	-2.75
1BX7	A	-2.38	-2.63	-2.74
1BXY	A	-1.07	-1.39	-1.75
1BYW	A	-1.38	-1.65	-2.31
1BZ4	A	-1.74	-1.78	-2.25
1C0F	S	-1.72	-2.42	-3.27
1C1Y	B	-1.40	-2.01	-2.16
1C25	A	-2.19	-4.13	-4.30
1C4P	A	-2.43	-3.10	-3.37
1C4R	A	-1.28	-1.87	-2.82
1C4Z	D	-1.16	-1.57	-1.05
1C6V	X	-1.59	-1.68	-1.59
1C9O	A	-1.04	-1.20	-1.32
1CC7	A	-1.09	-1.26	-1.65
1CDZ	A	-1.14	-1.49	-1.52
1CSK	A	-0.99	-0.90	-1.05
1D0Q	A	-2.30	-2.66	-2.89
1DTD	B	-1.03	-1.50	-2.03
1EAY	C	-1.51	-1.23	-1.30
1F94	A	-1.77	-1.85	-2.13
1FCC	C	-1.49	-1.48	-1.56

mean		-1.56	-1.93	-2.23

The Z-scores in the "Debye" column were calculated solely using the likelihood derived from the two-body model. The Z-scores in the "Debye+TorusDBN" column also include the prior distribution derived from Torus-DBN

In the second part of this experiment, we also incorporated a probabilistic model of the structure of proteins as a prior. This model, called TorusDBN, was previously developed in our group [21] and evaluates the probability of observing a certain sequence of ϕ and ψ angles for a given amino acid sequence. TorusDBN is a model of the *local *structure of proteins; non-local interactions such as hydrogen bonds or the formation of a hydrophobic core are not part of this model.

**Figure 8:**
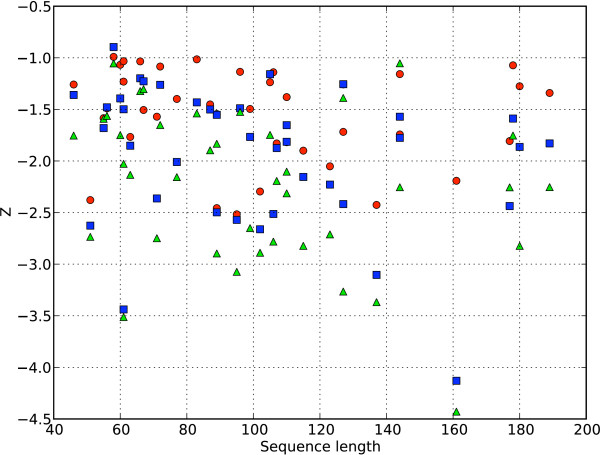
**Decoy recognition Z-scores for CRYSOL and the two-body model versus protein length**. For each of the proteins in the set, the Z-scores (*Y*-axis) are plotted against the length of the protein (*X*-axis). Red spheres: using CRYSOL. Blue squares: using the likelihood according to the two-body model. Green triangles: using the likelihood according to the two-body model and the TorusDBN prior. More detailed information is presented in Table 2.

Including the TorusDBN prior in the definition of the posterior probabilities leads to a clear improvement in decoy recognition; the average Z-score was enhanced by 16%. As illustrated in Figure 8, there is no correlation between protein size and Z-scores; the performance stays constant over a wide range of protein sizes.

## Conclusions

We have demonstrated that it is possible to obtain accurate SAXS curves from coarse-grained protein structures and matching estimated form factors without the use of *ad hoc *correction factors. We obtained point estimates of the form factors and assessed their performance for a diverse set of proteins; the resulting SAXS curves are on par with the current state-of-the-art program CRYSOL, at least up to scattering vector lengths of 0.75 Å^-1 ^(see Methods). As a further validation of this model, we used a comparison of the Z-scores for a set of protein decoys based on the SAXS curves generated by CRYSOL and by our coarse-grained Debye method, respectively. Again, the performance was excellent. Including prior information from a probabilistic model of local protein structure [21] further improved the decoy recognition.

Before a rigorous incorporation of SAXS information in a fully probabilistic model for data driven structure prediction is possible, two additional developments are needed: a proper description of the hydration layer that surrounds the protein [29] and a probabilistic description of the experimental errors associated with a SAXS data acquisition experiment [15,16]. We are currently implementing such an approach in the PHAISTOS software package [32,33].

## Methods

### Protein data sets

Three protein data sets were used throughout this work: one for training of the form factors, another for validating the model, and finally a set of native structures and corresponding artificial decoys for the Z-score calculations. The training data set consists of 297 structures with lengths between 50 and 400 from the Top500 data set of high quality protein structures [34]. To ensure that CRYSOL and our program processed these structures in the same manner, structures with conflicting atoms or non-standard amino acids were excluded (selected structures can be found in additional file 1).

The estimated form factors were validated by calculating scattering curves for 50 proteins (see Table 1) extracted using the PISCES server [35]. These were randomly selected among an initial group of 81 proteins with low sequence similarity (below 25%) with those in the training set, a resolution better than 3 Å and an R-factor below 30%.

The decoy set used in the Z-score evaluation was generated by the structure prediction program TASSER [31]. This decoy set consists of 47 proteins, each with 1040 protein-like decoy structures with varying similarity to the native structure. The decoys are constructed from energy-minimized snapshots from molecular dynamics simulations using the AMBER force field [36]. A single protein from the set, [1CBP], was excluded from our Z-score evaluation, as this structure resulted in an input error when evaluated by the CRYSOL program. Six proteins were left out of the evaluation as they were also present in our training data set.

In all cases, the backbone and side chain centroids were calculated using all non-hydrogen atoms. The Cβ atoms were only included in the calculation of the backbone centroid.

### SAXS training data

Due to the lack of publicly available high-quality experimental data needed for the estimation of the form factors, artificial data curves were generated for high-resolution protein structures using the state-of-the-art program CRYSOL [24]. This program calculates the theoretical scattering curve from a given full-atom resolution structure using spherical harmonics expansions. CRYSOL was used to compute a simulated scattering curve including the vacuum and excluded volume scattering components, but without hydration layer contribution; the electron density of the solvent layer was set equal to that of the bulk solvent (i.e. CRYSOL was run with the command line "crysol/dro 0.0 inputfile.pdb"). For the scattering curve evaluations, an upper *q*-limit of 0.75 Å^-1 ^was chosen in order to be well within the expected valid resolution range for CRYSOL.

### Error model and q-binning

The range of the scattering momentum ranges from 0 to 0.75 Å^-1^, divided in 51 discretized bins. For each bin we sampled form factors from the posterior distribution to obtain the 21-dimensional form factor distributions. The intensity at a given bin was evaluated using the left-hand side *q*-value, starting at *q *= 0 in the first bin.

To account for the "experimental" error for the SAXS curves, a standard deviation σ_*q *_= *I*_*q*_*β *(with *β *= 0.3) has previously been used in the literature as a realistic estimate [14]. Aiming to be more precise in the portion of the curve of primary interest in a structure prediction application - approximately between *q *= 0.1 and *q *= 0.5 Å^-1 ^[37] - we introduced a scaling factor (*q *+ *α*):

$$\sigma_{q}=I_{q}(q+\alpha )\beta$$

with *α *= 0.15 and *β *= 0.3. This is significantly stricter at mid *q*-range than the reference parameters.

### Posterior sampling

In order to explore the large parameter space efficiently, we used an optimized, maximum-likelihood based MCMC method implemented in the Muninn program [26]. In the MCMC method, we used the negative of the logarithm of the posterior probability as an energy. We employed a sampling scheme where the density of states is weighted according to the inverse cumulative density of states (1/*k *ensemble) [38]. Compared to standard MCMC methods, this ensures a more frequent generation of samples in the lower energy regions. Avoiding slow relaxation in the Markov chain also minimizes the risk of the chain getting trapped at a local minimum [26]; a problem often encountered in rough energy landscapes when using Metropolis techniques, as for example in simulated annealing.

An initial proposal value for the ${\overset{ª}{F}}_{q}$ vector was obtained by uniformly sampling its components from the interval [0, *f*_max_]. The value of *f*_max _was set to 40; well beyond the limit of the form factor components described in the literature [12]. The MCMC proposed new form factor vectors, ${{\overset{ª}{F}}^{\prime }}_{q}$, with a transition ${\overset{ª}{F}}_{q}\to {{\overset{ª}{F}}^{\prime }}_{q}$ in which a randomly chosen component *f *of the vector was re-sampled uniformly from the interval:

$$\left[\max\left\{0,f–m\right\},\;\;\min\{f_{\max},f+m\}\right]$$

where the width *m *was equal to 1.5. Border effects were taken into account in order to respect the detailed balance condition, which is

$$P({\overset{ª}{F}}_{q})P({\overset{ª}{F}}_{q}\to {{\overset{ª}{F}}^{\prime }}_{q})=P({{\overset{ª}{F}}^{\prime }}_{q})P({{\overset{ª}{F}}^{\prime }}_{q}\to {\overset{ª}{F}}_{q})$$

The transition probabilities, $P({\overset{ª}{F}}_{q}\to {{\overset{ª}{F}}^{\prime }}_{q})$, can be expressed as:

$$P({\overset{ª}{F}}_{q}\to {{\overset{ª}{F}}^{\prime }}_{q})=Q({\overset{ª}{F}}_{q}\to {\overset{ª}{F}}^{\prime }q)A({\overset{ª}{F}}_{q}\to {{\overset{ª}{F}}^{\prime }}_{q})$$

where $Q({\overset{ª}{F}}_{q}\to {{\overset{ª}{F}}^{\prime }}_{q})$ represents the probability of selecting ${{\overset{ª}{F}}^{\prime }}_{q}$ given ${\overset{ª}{F}}_{q}$ and $A({\overset{ª}{F}}_{q}\to {{\overset{ª}{F}}^{\prime }}_{q})$ is the acceptance probability of this transition. The selection probability implied by the local uniform proposal scheme is:

$$Q({\overset{ª}{F}}_{q}\to {{\overset{ª}{F}}^{\prime }}_{q})={\left(\min\{f_{\text{max}},f+m\}-\max\{0,f-m\}\right)}^{-1}$$

and according to the Metropolis-Hastings criterion [39], the following acceptance probability satisfies the detailed balance condition:

$$A({\overset{ª}{F}}_{q}\to {{\overset{ª}{F}}^{\prime }}_{q})=\min\left\{1,\;\frac{P({\overset{ª}{F^{\prime }}}_{q})Q({\overset{ª}{F^{\prime }}}_{q}\to {\overset{ª}{F}}_{q})}{P({\overset{ª}{F}}_{q})Q({\overset{ª}{F}}_{q}\to {\overset{ª}{F^{\prime }}}_{q})}\right\}$$

where $P({\overset{ª}{F}}_{q})$ is given below by Equations 7 and 8 for the TorusDBN independent and dependent cases, respectively.

### Point estimation of form factors

The MCMC procedure results in a set of samples from the posterior distribution. In order to obtain a point estimate, we calculated the centroid vector obtained from the set of sampled form factor vectors $\left\{{\overset{ª}{F}}_{q,1},\ldots ,{\overset{ª}{F}}_{q,T}\right\}$. The centroid vector ${\overset{ª}{F}}_{q,c}$ was defined as the one with the lowest Euclidean distance to all the other vectors in the set:

$${\overset{ª}{F}}_{q,c}=\;\underset{{\overset{ª}{F}}_{q,r}}{\arg\min}\sum_{t=1}{}^{T}\|{\overset{ª}{F}}_{q,r}-{\overset{ª}{F}}_{q,t}\|$$

where *T *is the number of samples.

### SAXS curve distance measures

A χ^2 ^measure was used to quantify the difference between two scattering curves. *S *was scaled by the "experimental" error that was used in the estimation of the form factors, thus evaluating the statistical quality of our reconstruction:

$$S\;=\sqrt{\frac{\Sigma_{q}{\left[(I_{q}-{I^{\prime }}_{q})/\sigma_{q}\right]}^{2}}{Q-1}}$$

where *Q *is the number of *q*-bins and σ_*q *_is the experimental error as described previously.

### Decoy recognition

The first decoy recognition experiment - which only uses the likelihood - is performed as follows. First, a SAXS curve *I *is calculated from the native structure using CRYSOL. For each decoy structure, a corresponding SAXS curve *I' *is calculated with the Debye formula using the two-body model. In this case, the posterior - which is simply equal to the likelihood *P*(*I *| *X*) - of the decoy *X *is calculated as:

(7) P(X|I)∝P(I|X)=P(I|I′)=∏q=1QN(Iq|I′q,|q)

where the product runs over all *q*-bins. For the comparison with CRYSOL, scattering curves were calculated from the decoys using CRYSOL instead of the two-body model.

In the second decoy recognition experiment, TorusDBN is included as a prior distribution, and amino acid information is explicitly included. In this case, the posterior becomes:

(8) $$\begin{array}{l} P(X|I,A)\propto P(I|X)P(X|A)\\ =\;\left\{\prod_{q=1}^{Q}N(I_{q}|{I^{\prime }}_{q},\sigma_{q})\right\}P(\overset{ª}{\phi },\overset{ª}{\psi }|A) \end{array}$$

where $\overset{ª}{\phi }$ and $\overset{ª}{\psi }$ are the backbone angles of the decoy and *A *is the amino acid sequence.

The Z-score for a given decoy set was calculated as the difference between the energy (defined as the negative of the logarithm of the posterior) of the native structure *E*(*X*_*N*_) and the average energy $\overset{ª}{E}$ of all structures in the decoy set, divided by the standard deviation *σ *of the energies:

$$Z=\frac{E(X_{N})-\overset{ª}{E}}{\sigma }$$

### TorusDBN prior

TorusDBN is a probabilistic model of the local structure of proteins, formulated as a dynamic Bayesian network. It can be considered as a probabilistic alternative to the well known fragment libraries, It allows sampling of plausible protein conformations in continuous space, and it can assign a probability density value to a given sequence of ϕ and ψ angles. For the prior, we used:

$$P(X|A)=P(\overset{ª}{\phi },\overset{ª}{\psi }|A)\propto P(\overset{ª}{\phi },\overset{ª}{\psi },A)$$

where $\overset{ª}{\phi }$ and $\overset{ª}{\psi }$ are the ϕ and ψ angle sequences, respectively. The calculation of $P(\overset{ª}{\phi },\overset{ª}{\psi },A)$ from TorusDBN is straightforward using the forward algorithm [21]. We used the model that is described in [21] with default parameters.

### Computational efficiency

The naive implementation of the Debye formula (Equation 2) leads to a computational complexity of *O*(*M*^2^), where *M *is the number of scatterers in the structure under evaluation. Our coarse-grained approach reduces *M *by representing several atoms by one scattering body (a dummy atom). Each of the dummy atoms contains an average of *k *atoms, thus lowering the execution time by a constant factor of *k*^2^, replacing *O*(*M*^2^) with $O\left({\left(\frac{M}{k}\right)}^{2}\right)$.

The exact value of *k *is obviously dependent on the primary sequence of the protein. For both training and validation sets, employing a dummy atom for the backbone and one for the side chain leads to an average *k *of 4.24. This means that each dummy atom contains on average 4.24 non-hydrogen atoms, leading to an increase in speed of *k*^2^≃18. Using only one dummy atom to describe a complete amino acid results in *k*≃7.8 atoms, allowing for a *k*^2^≃60 times faster execution. The absolute running time for a single scattering evaluation is approximately 30 ms for a 129 residues protein ([6LYZ]), using the two-body model and a standard desktop computer (AMD Athlon X2 5200+). Absolute running time comparisons with other programs are obviously unfair, since for a single evaluation the overhead introduced by the file system and the operative system is considerable. This said, our approach is significantly faster than CRYSOL [24](~786 ms).

## Availability

The point estimates of the form factor vectors for all bins are available as supplementary information (for the one-body model in additional file 2 and the two-body model in additional file 3).

## Authors' contributions

CA and KS contributed equally to this work. JFB provided the Muninn method, and assisted in its application. JFB and TH conceived the study. All authors read and approved the final manuscript.

## Supplementary Material

Additional file 1 **Data set of selected protein structures from the Top500 data set **[34]** used in the form factor estimation**. Each column contains PDB identifier, primary sequence length and radius of gyration calculated from the atomic structure. Click here for file

Additional file 2 **Scattering form factor centroids for the one-body model**. Form factor centroids for each amino acid in the *q*-range [0, 0.750] Å^-1^. Click here for file

Additional file 3 **Scattering form factors for the two-body model**. Form factor centroids for the generic backbone component and each amino acid in the *q*-range [0, 0.750] Å^-1^. An asterisk is used to mark the residues where the form factor includes both the backbone and side chain scatterers. Click here for file
