# Supplementary material for: Calculation of accurate small angle X-ray scattering curves from coarse-grained protein models
Source: BMC Bioinformatics. 2010 Aug 18;11:429. doi: 10.1186/1471-2105-11-429 (PMC2931518; doi:10.1186/1471-2105-11-429)
Supplement: Additional file 1 — Data set of selected protein structures from the Top500 data set [34] used in the form factor estimation. Each column contains PDB identifier, primary sequence length and radius of gyration calculated from the atomic structure. [file 1471-2105-11-429-S1.PDF]

Table 1: Protein structures used in the form factor estimation

PDB	Length	Rg	PDB	Length	Rg
1A92	50	10.63	1HCR	52	7.03
1RB9	52	5.60	2FDN	55	5.63
1TGS	56	6.33	1BPI	58	6.54
1FXD	58	5.86	1NKD	59	8.05
1TGX	60	7.08	1TUD	60	6.29
1VIE	60	6.26	1FAS	61	6.72
2IGD	61	6.92	1ISU	62	6.21
3EBX	62	6.80	1A1Y	63	6.51
1BF4	63	6.57	2SN3	65	6.47
1C90	66	6.35	1MSI	66	6.13
1PCF	66	8.31	1B3A	67	7.29
1VFY	67	7.27	1BBZ	68	6.25
1CTF	68	6.45	1C75	71	6.17
1CC8	72	6.51	1DP7	76	7.43
1NDD	76	6.64	1TIF	76	7.94
1VCC	77	7.09	1KP6	79	6.94
1BDO	80	6.94	1UGI	82	7.09
3HTS	82	7.07	451C	82	6.71
3EIP	84	7.30	1AAY	85	10.05
1B0Y	85	6.82	1OPD	85	6.78
2BOP	85	7.85	1ABA	87	7.41
1GVP	87	8.41	1CYO	88	7.36
1AY7	89	6.92	1CTJ	89	6.82
1FNA	91	7.73	1RZL	91	6.89
1LMB	92	7.86	1DFU	94	7.46
1MOL	94	7.98	1C5E	95	7.20
1FLT	95	10.48	1FLT	95	10.48
1RGE	96	7.18	2CBP	96	7.13
1CZP	98	6.93	2ACY	98	7.41
1BM8	99	7.24	1DIF	99	7.82
1PLC	99	7.15	1PSR	100	8.17
3EZM	101	11.20	3VUB	101	7.77
1SVF	102	14.69	1IIB	103	7.42
3CHB	103	7.72	5CYT	103	7.37
1CMB	104	8.50	1ERV	105	7.29
1KPT	105	7.19	256B	106	8.40
7FD1	106	7.16	1FKJ	107	7.71
2PVB	107	7.18	2SPC	107	17.20
3CYR	107	8.37	1A2P	108	7.84
1BKR	108	7.41	2TRX	108	7.33
1LKK	109	7.68	2MCM	112	7.78
2TGI	112	10.67	1MFI	114	8.32
2HMZ	114	7.75	1EVH	116	7.84

Table 1: Protein structures used in the form factor estimation

PDB	Length	Rg	PDB	Length	Rg
1SWU	116	8.67	1DPT	117	8.53
1POA	118	8.28	2MHR	118	8.02
1DHN	121	9.07	2SAK	121	9.19
1FLM	122	8.21	1WHI	122	8.03
2CUA	122	7.87	1BGF	124	8.79
1BQK	124	7.63	7RSA	124	8.35
2BBK	125	8.08	3PYP	125	7.66
1BFG	126	7.75	1RIE	127	8.10
1TTB	127	8.61	1NWP	128	7.94
3CHY	128	7.72	1CPQ	129	9.00
4LZT	129	8.10	1BBH	131	9.19
1C52	131	7.94	1HMT	131	8.17
1IFC	131	8.12	2LIS	131	9.59
1KUH	132	7.86	1NFN	132	9.74
1VSR	134	8.81	1C1L	135	8.15
1EUW	136	9.76	1STN	136	8.47
2END	137	9.10	1KVE	140	7.86
1AQZ	142	8.62	1BAB	142	8.45
1FLP	142	8.38	1TFE	142	10.44
1CXQ	143	8.93	1A3A	144	8.12
1M6P	146	8.73	1MBA	146	8.38
1AKR	147	8.00	2HBG	147	8.41
1OSA	148	12.64	2TNF	148	9.70
1BJ7	150	8.24	1NPK	150	8.66
1A6M	151	8.74	1ELK	153	8.59
1GDJ	153	8.63	1NBC	155	8.55
2RN2	155	9.04	1DPS	156	9.79
3PVI	156	10.40	1HFC	157	8.49
1VHH	157	8.55	1HKA	158	8.90
1RA9	159	8.88	1D7P	160	8.70
119L	162	9.64	1A73	162	11.42
1PHN	162	10.46	2CPL	164	8.37
1MUG	165	8.48	1C02	166	9.50
1CJW	166	8.74	5P21	166	8.68
1MGT	169	8.73	1RCF	169	8.40
1CCZ	171	13.16	1KOE	172	8.46
1CV8	173	9.11	1AMM	174	9.55
1AQB	175	9.21	1YTB	180	11.71
1BEH	183	8.81	1ATZ	184	8.58
1ERX	184	8.84	1IDO	184	8.77
153L	185	8.79	1XNB	185	8.64
2GAR	188	9.39	1BK7	190	9.41
2PTH	193	9.08	1NKR	195	11.12

Table 1: Protein structures used in the form factor estimation

PDB	Length	Rg	PDB	Length	Rg
1QHV	195	9.79	1CEX	197	8.80
1CHD	198	8.82	1QQ4	198	8.73
1VCA	199	13.78	1IAB	200	9.59
1NOX	200	11.05	1ATL	201	9.27
1BSM	201	10.10	1YAC	204	9.77
2HFT	205	13.10	1UCH	206	9.31
1BS9	207	8.80	1THV	207	9.46
1CKE	212	10.19	1WAB	212	9.45
2AYH	214	9.29	1C1K	217	10.97
1VFR	217	11.21	1ZIN	217	9.88
6GSV	217	10.15	1AUO	218	9.32
1A2Z	220	9.72	1A7S	221	9.52
1C3W	222	10.16	2NLR	222	9.12
1BYI	224	9.80	1MUN	225	10.43
4EUG	225	9.78	2TPS	226	9.41
1AMF	231	10.41	1DLF	233	10.38
1QB7	236	10.83	1NLS	237	9.91
1DVJ	239	9.85	1GDO	240	9.56
1QHF	240	10.07	1QNJ	240	9.56
1B5E	241	10.77	1QL0	241	9.64
1AGJ	242	10.15	1CYD	242	10.10
2BAA	243	9.86	1AH7	245	10.31
1QQ5	245	10.18	1TPH	245	9.89
1D2N	246	11.70	1MRJ	247	10.20
1QTS	247	11.58	1A28	249	10.41
1MML	251	11.89	1B16	254	10.37
2CBA	258	10.09	1QGI	259	10.55
1QSG	259	10.45	1ES5	260	10.20
1CL8	261	12.05	1ARB	263	9.56
1AK0	264	10.18	1SML	266	10.43
1H2R	267	11.50	1AKO	268	10.23
1CB0	268	10.28	1GCI	269	9.38
1NZY	269	11.42	2DRI	271	11.16
1DCI	275	11.55	1BRT	277	10.00
1TOA	277	11.13	1B8O	280	10.41
1AQU	281	10.76	1QTW	285	10.52
1TML	286	10.38	1NAR	289	10.58
1AMP	291	9.95	1BF6	291	10.21
19HC	292	13.56	1C3D	294	10.44
1HCL	294	11.40	1A4I	295	11.47
1FNC	296	11.30	1FTR	296	12.05
7A3H	300	10.09	2POR	301	11.77
1TAX	302	10.59	1CEQ	304	11.19

Table 1: Protein structures used in the form factor estimation

PDB	Length	Rg	PDB	Length	Rg
1MLA	305	10.67	7ATJ	305	11.09
2CTC	307	10.41	1DOZ	309	11.45
1GCA	309	11.89	1CIP	316	12.02
1CVL	316	10.60	1TCA	317	10.45
1IXH	321	11.59	1AXN	323	12.34
1B4K	326	11.84	1BDM	327	11.32
1A8E	329	11.34	4PGA	330	11.68
1NIF	333	12.06	1GD1	334	11.69
1CZF	335	11.46	1ARU	336	11.22
1UXY	340	11.70	1XIK	340	12.11
1GUQ	347	11.86	3PTE	347	10.93
1BG6	349	11.38	1B6A	355	11.70
2BBK	355	11.45	1URO	357	11.27
1DOS	358	12.78	1QCX	359	11.28
1CEM	363	10.83	1CNZ	363	12.01
1MOQ	366	11.62	1HTR	372	11.53
2NAC	374	12.52	3BTO	374	11.92
3SIL	379	11.16	1QH4	380	12.69
1BD0	381	13.84	1BS0	383	12.54
2QWC	388	11.29			
