# Supplementary material for: Calculation of accurate small angle X-ray scattering curves from coarse-grained protein models
Source: BMC Bioinformatics. 2010 Aug 18;11:429. doi: 10.1186/1471-2105-11-429 (PMC2931518; doi:10.1186/1471-2105-11-429)
Supplement: Additional file 2 — Scattering form factor centroids for the one-body model. Form factor centroids for each amino acid in the q-range [0, 0.750] Å-1. [file 1471-2105-11-429-S2.PDF]

q	ALA	ARG	ASN	ASP	CYS	GLN	GLU	GLY	HIS	ILE	LEU	LYS	MET	PHE	PRO	SER	THR	TRP	TYR	VAL
0.000	8.983	23.527	20.007	20.157	18.588	19.176	19.438	10.084	21.165	6.196	6.012	11.445	16.351	8.908	9.026	14.157	13.012	15.423	14.108	7.008
0.015	8.980	23.516	19.999	20.150	18.589	19.173	19.434	10.083	21.162	6.191	6.015	11.463	16.362	8.907	9.035	14.162	13.026	15.406	14.109	7.004
0.030	8.980	23.482	19.991	20.143	18.615	19.167	19.430	10.081	21.151	6.191	6.013	11.484	16.391	8.909	9.059	14.165	13.044	15.429	14.118	7.007
0.045	8.982	23.431	19.965	20.115	18.663	19.159	19.418	10.070	21.126	6.193	6.017	11.531	16.451	8.921	9.098	14.169	13.073	15.471	14.128	7.007
0.060	8.977	23.359	19.946	20.104	18.703	19.167	19.410	10.072	21.107	6.177	6.020	11.605	16.522	8.887	9.160	14.179	13.119	15.470	14.137	7.000
0.075	8.973	23.285	19.933	20.086	18.772	19.177	19.388	10.056	21.090	6.162	6.024	11.710	16.605	8.852	9.220	14.184	13.170	15.501	14.139	6.999
0.090	8.964	23.210	19.916	20.090	18.831	19.202	19.382	10.041	21.059	6.130	6.012	11.857	16.678	8.789	9.320	14.192	13.232	15.505	14.133	6.984
0.105	8.939	23.165	19.940	20.160	18.848	19.234	19.397	10.047	21.060	6.061	5.984	12.059	16.742	8.650	9.483	14.216	13.277	15.434	14.088	6.952
0.120	8.902	23.140	19.995	20.284	18.828	19.267	19.422	10.068	21.075	5.982	5.935	12.321	16.841	8.436	9.726	14.259	13.331	15.312	14.001	6.893
0.135	8.859	23.127	20.110	20.440	18.761	19.325	19.507	10.108	21.162	5.902	5.852	12.612	16.967	8.164	10.030	14.358	13.363	15.127	13.876	6.818
0.150	8.823	23.103	20.311	20.476	18.714	19.437	19.569	10.168	21.320	5.848	5.774	12.956	17.108	7.884	10.294	14.518	13.338	14.884	13.749	6.790
0.165	8.831	23.076	20.539	20.302	18.714	19.650	19.546	10.141	21.472	5.829	5.721	13.368	17.254	7.628	10.494	14.676	13.292	14.790	13.670	6.852
0.180	8.943	23.389	20.588	20.275	18.804	19.577	19.297	10.082	21.726	5.688	5.783	13.810	17.199	7.385	10.423	14.671	13.288	14.607	13.923	6.964
0.195	8.967	23.717	20.370	20.183	18.797	19.366	19.374	10.199	22.191	5.570	5.686	14.125	16.991	7.300	9.967	14.937	13.357	14.119	14.321	7.184
0.210	9.042	23.798	20.572	20.101	18.670	18.959	20.016	10.291	22.256	5.551	5.481	13.889	17.223	7.293	9.845	15.019	13.728	13.912	14.380	7.331
0.225	9.049	23.609	20.796	20.341	18.563	19.074	20.079	10.530	22.056	5.405	5.466	13.887	17.413	7.493	10.009	15.224	13.522	13.622	14.320	7.511
0.240	9.213	23.289	20.454	20.410	18.613	19.204	19.987	10.724	22.194	5.048	5.571	13.838	18.040	7.636	10.238	15.797	13.315	13.914	14.146	7.636
0.255	9.444	22.735	20.612	20.356	18.758	19.736	19.869	11.095	22.209	4.925	5.791	13.597	18.760	7.597	10.536	15.528	13.388	14.824	13.894	7.641
0.270	9.695	22.461	21.085	20.519	18.740	19.952	19.712	11.680	22.229	5.137	6.129	13.461	18.699	7.856	10.776	15.035	13.139	15.986	13.651	7.479
0.285	10.014	22.591	21.292	20.668	18.608	20.022	19.412	11.957	22.483	5.409	6.368	13.750	18.508	7.649	10.973	14.996	12.985	16.097	13.574	7.631
0.300	10.495	22.668	21.243	20.909	18.347	20.159	19.204	11.758	22.616	5.535	6.655	14.127	18.185	7.771	11.296	15.080	13.447	14.860	13.885	8.017
0.315	10.991	22.535	21.245	21.186	18.010	20.498	19.536	11.389	22.931	5.674	6.816	14.207	17.774	8.396	11.651	15.046	14.065	13.185	14.242	8.758
0.330	11.357	22.339	21.371	21.421	17.955	20.890	20.356	11.183	23.695	6.235	6.813	14.099	17.384	9.104	11.453	15.009	14.376	11.716	14.239	9.795
0.345	11.584	22.061	21.440	21.670	18.229	21.405	21.185	11.169	24.445	7.201	6.851	14.176	17.188	10.111	10.847	15.214	14.254	10.927	13.691	10.783
0.360	11.825	21.798	21.480	21.852	18.566	22.003	21.868	11.244	24.758	8.314	7.147	14.352	17.394	11.322	10.227	15.504	14.047	11.104	12.764	11.375
0.375	12.027	21.689	21.526	21.894	18.718	22.339	22.339	11.298	24.793	9.102	7.435	14.540	18.271	12.263	10.002	15.853	14.027	11.717	12.196	11.736
0.390	12.154	21.878	21.623	21.932	18.672	22.297	22.462	11.282	24.873	9.500	7.555	14.813	19.525	12.850	10.376	16.039	14.219	12.012	12.596	12.183
0.405	12.258	22.426	21.773	22.092	18.648	21.977	22.217	11.148	24.895	9.695	7.571	15.402	20.624	13.300	11.022	16.036	14.357	11.155	13.387	12.829
0.420	12.320	23.333	21.964	22.399	18.603	21.534	21.673	11.024	25.171	9.903	7.923	16.334	21.199	13.734	11.757	15.941	14.328	9.458	13.997	13.611
0.435	12.388	24.332	22.074	22.772	18.506	21.015	21.224	10.897	25.710	10.066	8.423	17.260	20.906	13.560	12.274	15.798	14.206	7.704	14.203	14.305
0.450	12.578	25.094	22.175	23.098	18.326	20.724	21.121	10.736	26.589	10.084	8.957	17.929	20.026	12.778	12.504	15.679	14.237	6.901	14.064	14.871
0.465	12.995	25.463	22.154	23.348	18.165	20.791	21.410	10.598	27.522	10.175	9.780	18.201	19.046	11.570	12.168	15.697	14.551	7.018	13.738	15.272
0.480	13.734	25.314	21.949	23.483	17.846	21.207	21.975	10.502	28.195	10.351	11.359	18.122	17.236	10.345	11.294	15.787	14.986	8.764	13.068	15.731
0.495	14.665	24.749	21.577	23.520	17.466	21.629	22.598	10.765	28.676	10.296	14.696	17.900	14.246	9.393	10.165	15.942	15.496	13.482	11.615	16.159
0.510	15.489	23.664	20.780	23.156	16.763	21.307	23.048	11.365	28.570	9.649	18.273	17.847	9.988	8.415	8.712	16.096	15.843	19.269	9.564	16.297
0.525	16.160	22.416	20.301	22.587	15.608	20.051	23.382	12.132	28.170	9.499	20.940	18.183	7.757	7.305	7.580	16.476	16.015	21.923	7.980	15.840
0.540	16.718	20.920	20.372	21.905	14.329	18.092	23.623	12.822	27.608	10.376	22.978	18.922	6.642	6.000	6.886	17.026	15.919	22.435	6.978	15.165
0.555	17.124	18.782	20.847	21.051	12.927	15.184	23.800	13.387	26.772	13.195	24.566	19.822	5.209	4.842	6.839	17.468	15.266	21.298	6.159	14.598
0.570	17.444	16.209	21.396	20.036	11.837	12.035	23.762	13.643	25.775	17.873	25.923	20.604	2.968	4.008	7.611	17.646	14.350	19.959	5.445	14.341
0.585	17.888	12.826	21.555	19.140	11.032	7.940	23.533	13.419	24.295	20.246	27.185	21.047	2.245	3.307	8.660	17.607	13.437	18.572	4.800	14.227
0.600	18.489	11.902	21.175	18.360	10.836	5.693	23.286	12.834	22.154	22.079	28.188	21.224	1.991	2.879	9.679	17.403	12.759	17.664	4.576	14.105

q	ALA	ARG	ASN	ASP	CYS	GLN	GLU	GLY	HIS	ILE	LEU	LYS	MET	PHE	PRO	SER	THR	TRP	TYR	VAL
0.615	19.253	13.965	20.329	17.337	11.276	4.756	22.977	12.109	18.472	23.991	29.006	21.551	2.118	2.577	10.361	17.152	12.454	16.629	4.908	13.555
0.630	20.102	18.271	19.272	15.073	11.995	4.208	22.223	11.353	12.441	26.021	29.474	22.141	2.277	2.240	10.929	16.978	12.410	16.511	6.301	12.570
0.645	21.142	22.799	17.612	5.113	12.337	3.852	21.597	11.304	6.818	27.353	28.914	22.824	2.126	1.392	12.476	17.426	12.207	20.393	9.286	11.273
0.660	21.720	23.039	16.274	3.168	8.550	4.878	24.394	12.640	12.463	24.926	27.484	3.448	10.288	0.936	12.961	17.219	11.205	4.087	4.718	15.946
0.675	22.053	23.160	15.897	1.868	6.480	6.599	24.714	13.126	14.732	23.490	26.697	0.275	14.905	0.802	11.967	17.176	11.326	1.997	3.336	16.694
0.690	22.270	22.848	16.406	1.532	5.353	9.751	24.467	13.534	15.732	22.288	26.162	0.244	16.764	0.782	10.695	16.856	11.496	1.997	3.177	16.493
0.705	22.364	22.516	17.171	1.426	5.284	13.656	24.395	13.999	16.424	20.421	25.505	0.283	17.967	0.973	9.432	16.431	11.751	2.751	4.055	16.023
0.720	22.311	22.879	18.119	0.673	8.562	15.659	23.875	14.978	20.007	7.818	24.661	0.241	19.062	1.143	7.469	16.389	12.260	3.888	9.183	15.302
0.735	22.147	22.537	18.614	0.841	14.915	13.750	22.830	14.827	21.604	2.019	24.557	0.283	13.458	1.450	6.227	14.899	12.513	6.638	15.973	14.112
0.750	21.889	21.659	18.284	1.174	19.476	10.968	21.996	14.276	21.131	1.580	24.669	0.248	9.862	1.505	6.212	13.158	12.333	10.273	18.422	13.382
