# Supplementary material for: Calculation of accurate small angle X-ray scattering curves from coarse-grained protein models
Source: BMC Bioinformatics. 2010 Aug 18;11:429. doi: 10.1186/1471-2105-11-429 (PMC2931518; doi:10.1186/1471-2105-11-429)
Supplement: Additional file 3 — Scattering form factors for the two-body model. Form factor centroids for the generic backbone component and each amino acid in the q-range [0, 0.750] Å-1. An asterisk is used to mark the residues where the form factor includes both the backbone and side chain scatterers. [file 1471-2105-11-429-S3.PDF]

q	BB	ALA*	ARG	ASN	ASP	CYS	GLN	GLU	GLY*	HIS	ILE	LEU	LYS	MET	PHE	PRO	SER	THR	TRP	TYR	VAL
0.000	5.792	8.982	17.724	14.211	14.357	12.804	13.373	13.644	10.085	15.369	0.403	0.244	5.651	10.567	3.123	3.229	8.363	7.223	9.634	8.322	1.213
0.015	5.791	8.978	17.724	14.219	14.359	12.811	13.373	13.652	10.087	15.369	0.406	0.254	5.660	10.585	3.131	3.236	8.372	7.233	9.647	8.327	1.215
0.030	5.818	8.969	17.702	14.217	14.345	12.801	13.358	13.651	10.096	15.350	0.389	0.246	5.657	10.610	3.126	3.232	8.366	7.236	9.648	8.321	1.202
0.045	5.892	8.955	17.634	14.185	14.291	12.755	13.297	13.618	10.110	15.291	0.334	0.216	5.620	10.607	3.083	3.189	8.323	7.207	9.617	8.272	1.145
0.060	6.016	8.937	17.519	14.126	14.200	12.673	13.207	13.548	10.120	15.195	0.240	0.152	5.552	10.576	2.999	3.099	8.243	7.145	9.549	8.190	1.051
0.075	6.125	8.920	17.420	14.108	14.131	12.625	13.151	13.500	10.128	15.122	0.189	0.131	5.518	10.559	2.924	3.017	8.182	7.104	9.511	8.135	0.979
0.090	6.265	8.904	17.292	14.080	14.041	12.548	13.084	13.415	10.129	15.033	0.132	0.094	5.486	10.496	2.817	2.907	8.097	7.038	9.439	8.065	0.887
0.105	6.406	8.880	17.171	14.073	13.955	12.483	13.038	13.324	10.131	14.953	0.120	0.087	5.487	10.403	2.701	2.800	8.019	6.971	9.358	8.012	0.800
0.120	6.554	8.854	17.063	14.086	13.895	12.387	13.037	13.232	10.135	14.892	0.106	0.074	5.529	10.300	2.548	2.723	7.958	6.894	9.235	7.962	0.704
0.135	6.729	8.838	16.926	14.083	13.806	12.265	13.066	13.107	10.142	14.846	0.078	0.053	5.571	10.187	2.337	2.622	7.915	6.777	9.040	7.894	0.594
0.150	6.930	8.840	16.690	14.063	13.598	12.181	13.158	12.976	10.132	14.777	0.071	0.048	5.573	10.092	2.060	2.418	7.878	6.616	8.813	7.828	0.504
0.165	7.162	8.884	16.432	14.006	13.304	12.154	13.266	12.792	10.070	14.647	0.078	0.061	5.496	10.029	1.689	2.119	7.718	6.395	8.694	7.732	0.441
0.180	7.368	9.014	16.582	14.221	13.239	11.948	13.055	12.384	10.033	14.781	0.047	0.044	5.647	9.986	1.403	1.848	7.442	6.407	8.621	7.582	0.350
0.195	7.521	9.125	17.042	14.145	12.997	11.711	12.436	12.548	10.198	14.983	0.052	0.047	5.703	9.886	1.464	1.495	7.541	6.483	8.700	7.385	0.331
0.210	7.675	9.331	17.065	14.310	12.708	11.441	12.176	13.209	10.364	14.809	0.053	0.044	5.718	9.876	1.577	1.727	7.575	6.462	8.588	7.070	0.222
0.225	7.867	9.525	16.703	14.020	12.900	11.256	12.302	13.196	10.522	14.427	0.050	0.039	5.842	9.891	1.795	2.000	7.500	6.215	8.193	6.744	0.143
0.240	8.031	9.714	16.793	13.572	12.835	11.010	12.513	12.988	10.798	14.329	0.040	0.037	5.747	9.908	1.962	2.205	7.900	5.974	7.711	6.610	0.128
0.255	8.232	9.836	16.536	13.610	12.656	10.851	12.999	12.934	11.101	13.971	0.036	0.039	5.603	9.976	1.850	2.394	7.607	5.667	7.668	6.567	0.103
0.270	8.458	9.880	16.213	13.648	12.582	10.745	13.317	12.937	11.405	13.662	0.051	0.057	5.566	9.742	1.553	2.364	7.104	5.533	7.825	6.394	0.099
0.285	8.734	10.022	15.934	13.379	12.391	10.406	13.383	12.525	11.473	13.808	0.043	0.042	5.584	9.379	1.177	2.243	6.811	5.734	8.353	6.235	0.066
0.300	9.066	10.353	15.636	12.895	12.286	9.744	13.116	12.007	11.465	13.986	0.047	0.045	5.262	8.802	0.990	2.082	6.546	5.920	8.058	6.417	0.068
0.315	9.416	10.743	15.348	12.423	12.120	9.064	12.806	11.822	11.492	14.110	0.054	0.051	4.776	7.966	1.081	1.919	6.260	5.898	7.043	6.631	0.095
0.330	9.740	11.025	15.208	12.127	11.893	8.743	12.620	11.920	11.494	14.388	0.056	0.048	4.518	7.051	1.319	1.666	6.008	5.551	6.200	6.450	0.133
0.345	10.019	11.209	15.235	11.930	11.832	8.685	12.571	12.022	11.462	14.568	0.077	0.055	4.506	6.505	1.700	1.350	5.848	5.073	6.101	5.948	0.200
0.360	10.265	11.408	15.404	11.818	11.861	8.661	12.431	12.022	11.400	14.581	0.072	0.046	4.554	6.416	2.073	1.091	5.742	4.728	6.662	5.521	0.190
0.375	10.485	11.565	15.722	11.730	11.910	8.579	12.132	11.886	11.337	14.424	0.096	0.055	4.604	6.688	2.171	1.090	5.672	4.556	7.372	5.548	0.210
0.390	10.717	11.641	16.156	11.546	11.930	8.450	11.726	11.482	11.297	14.103	0.086	0.048	4.731	7.251	2.032	1.237	5.565	4.502	7.849	6.028	0.180
0.405	10.965	11.659	16.681	11.287	11.925	8.274	11.276	10.877	11.285	13.630	0.124	0.069	5.025	7.866	1.871	1.410	5.407	4.452	7.529	6.567	0.235
0.420	11.211	11.721	17.097	11.020	11.896	8.121	10.780	10.324	11.297	13.251	0.114	0.062	5.326	8.308	1.835	1.449	5.186	4.363	6.676	6.815	0.208
0.435	11.442	11.837	17.230	10.840	11.780	7.931	10.174	10.110	11.402	13.243	0.139	0.075	5.369	8.239	1.869	1.408	4.887	4.181	5.881	6.696	0.233
0.450	11.658	12.062	17.045	10.710	11.493	7.770	9.567	10.189	11.532	13.641	0.111	0.060	5.097	7.755	1.870	1.191	4.563	3.940	5.409	6.352	0.187
0.465	11.861	12.360	16.612	10.562	11.068	7.615	9.010	10.361	11.626	14.109	0.111	0.060	4.733	7.021	1.779	0.909	4.289	3.648	5.222	5.916	0.183
0.480	12.051	12.657	15.996	10.303	10.627	7.439	8.529	10.385	11.699	14.449	0.149	0.081	4.470	6.165	1.638	0.803	4.074	3.374	5.261	5.481	0.244
0.495	12.219	12.949	15.265	9.915	10.239	7.290	8.077	10.253	11.756	14.726	0.176	0.098	4.380	5.443	1.523	0.770	4.006	3.186	5.465	5.144	0.304
0.510	12.354	13.224	14.564	9.455	9.898	7.272	7.694	10.036	11.861	14.941	0.116	0.066	4.346	5.142	1.346	0.571	4.160	3.070	5.725	4.947	0.233
0.525	12.476	13.460	13.725	9.040	9.465	7.105	7.313	9.869	12.028	14.996	0.156	0.089	4.237	5.061	1.320	0.677	4.377	2.922	5.787	4.842	0.339
0.540	12.582	13.711	12.865	8.682	8.934	7.156	7.221	9.791	12.219	14.957	0.134	0.080	3.981	5.471	1.272	0.625	4.659	2.657	5.842	4.872	0.363
0.555	12.681	13.964	12.054	8.345	8.394	7.229	7.345	9.891	12.445	14.875	0.131	0.087	3.613	6.043	1.314	0.627	4.792	2.242	6.013	4.944	0.457
0.570	12.786	14.185	11.294	7.936	7.908	7.181	7.534	9.904	12.685	14.705	0.217	0.156	3.302	6.365	1.534	0.869	4.650	1.868	6.394	4.962	0.681
0.585	12.878	14.354	10.869	7.458	7.686	7.365	8.019	9.906	12.952	14.434	0.175	0.136	2.956	7.040	1.550	0.754	4.384	1.469	6.953	4.951	0.566
0.600	12.962	14.469	10.517	6.826	7.602	7.460	8.613	9.656	13.214	13.746	0.211	0.173	2.580	7.290	1.709	0.900	4.069	1.393	7.417	4.859	0.529

q	BB	ALA*	ARG	ASN	ASP	CYS	GLN	GLU	GLY*	HIS	ILE	LEU	LYS	MET	PHE	PRO	SER	THR	TRP	TYR	VAL
0.615	13.032	14.559	10.238	6.111	7.583	7.624	9.400	9.307	13.408	12.603	0.239	0.204	2.096	7.442	1.801	1.117	3.838	1.547	7.585	4.843	0.487
0.630	13.087	14.687	9.800	5.377	7.537	7.690	10.363	8.688	13.464	11.340	0.319	0.277	1.582	7.438	1.911	1.435	3.774	1.995	7.613	4.834	0.533
0.645	13.130	14.857	9.316	4.699	7.438	7.693	11.288	7.846	13.452	10.506	0.404	0.335	1.158	7.480	1.995	1.620	3.819	2.616	7.731	4.771	0.615
0.660	13.169	15.097	8.983	4.290	7.216	7.658	11.924	6.964	13.396	10.236	0.488	0.349	0.921	7.532	2.091	1.560	3.855	3.374	8.062	4.576	0.671
0.675	13.228	15.367	9.028	4.275	6.741	7.692	12.075	6.399	13.335	10.330	0.531	0.330	0.847	7.680	2.255	1.310	3.851	4.233	8.504	4.366	0.682
0.690	13.310	15.635	9.941	4.798	5.988	7.822	11.833	6.411	13.317	10.488	0.508	0.314	0.951	7.807	2.506	1.045	3.812	5.100	8.882	4.270	0.639
0.705	13.407	15.865	11.468	5.689	5.080	7.936	11.072	6.904	13.424	10.146	0.700	0.526	1.604	7.913	2.848	1.177	3.652	5.724	8.933	4.524	0.828
0.720	13.409	16.023	14.272	7.015	4.739	8.128	10.843	7.788	13.674	9.670	0.475	0.469	1.307	8.752	2.839	0.920	3.701	6.086	9.007	4.934	0.649
0.735	13.428	16.120	15.414	7.848	4.726	8.211	10.317	8.329	13.874	8.681	0.663	0.759	1.538	8.946	2.836	1.119	3.592	6.113	9.573	5.830	0.853
0.750	13.429	16.122	16.070	8.492	5.115	8.209	9.842	8.817	14.105	7.768	0.832	1.064	2.204	8.227	2.715	1.187	3.629	6.077	10.749	7.011	0.983
